# Three-dimensional Noninvasive Monitoring Iodine-131 Uptake in the Thyroid Using a Modified Cerenkov Luminescence Tomography Approach

**DOI:** 10.1371/journal.pone.0037623

**Published:** 2012-05-22

**Authors:** Zhenhua Hu, Xiaowei Ma, Xiaochao Qu, Weidong Yang, Jimin Liang, Jing Wang, Jie Tian

**Affiliations:** 1 School of Life Sciences and Technology, Xidian University, Xi’an, China; 2 Department of Nuclear Medicine, Xijing Hospital, Fourth Military Medical University, Xi’an, China; 3 Institute of Automation, Chinese Academy of Sciences, Beijing, China; National Institute of Health, United States of America

## Abstract

**Background:**

Cerenkov luminescence tomography (CLT) provides the three-dimensional (3D) radiopharmaceutical biodistribution in small living animals, which is vital to biomedical imaging. However, existing single-spectral and multispectral methods are not very efficient and effective at reconstructing the distribution of the radionuclide tracer. In this paper, we present a semi-quantitative Cerenkov radiation spectral characteristic-based source reconstruction method named the hybrid spectral CLT, to efficiently reconstruct the radionuclide tracer with both encouraging reconstruction results and less acquisition and image reconstruction time.

**Methodology/Principal Findings:**

We constructed the implantation mouse model implanted with a 400 *µ*Ci Na^131^I radioactive source and the physiological mouse model received an intravenous tail injection of 400 *µ*Ci radiopharmaceutical Iodine-131 (I-131) to validate the performance of the hybrid spectral CLT and compared the reconstruction results, acquisition, and image reconstruction time with that of single-spectral and multispectral CLT. Furthermore, we performed 3D noninvasive monitoring of I-131 uptake in the thyroid and quantified I-131 uptake *in vivo* using hybrid spectral CLT. Results showed that the reconstruction based on the hybrid spectral CLT was more accurate in localization and quantification than using single-spectral CLT, and was more efficient in the *in vivo* experiment compared with multispectral CLT. Additionally, 3D visualization of longitudinal observations suggested that the reconstructed energy of I-131 uptake in the thyroid increased with acquisition time and there was a robust correlation between the reconstructed energy versus the gamma ray counts of I-131 (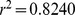). The *ex vivo* biodistribution experiment further confirmed the I-131 uptake in the thyroid for hybrid spectral CLT.

**Conclusions/Significance:**

Results indicated that hybrid spectral CLT could be potentially used for thyroid imaging to evaluate its function and monitor its treatment for thyroid cancer.

## Introduction

Cerenkov radiation was first observed as early as 1934, which was named after Russian scientist Pavel Alekseyevich Cherenkov, the 1958 Nobel Physics Laureate who first characterized it rigorously [1]. If the speed of a charged and high-energy particle is faster than the speed of light in a dielectric medium, visible and near-infrared photons are emitted [1], [2], [3]. At that time, it was not applied to biomedical imaging since the optical signals were too weak to be detected due to the limitation of the optical detection devices.

With the development of the highly sensitive charge-coupled device (CCD) camera, Cerenkov radiation has been applied to *in vivo* optical imaging of small living animals in recent years [4]–[16]. Robertson *et al*. first presented *in vivo* Cerenkov luminescence imaging (CLI) in mice using a highly sensitive CCD camera [4]. Spinelli *et al*. presented a detailed model of the Cerenkov radiation spectrum considering the positron energy spectrum to quantify the amount of light emission [5]. *In vivo* optical imaging of radiotracers that emit charged particles such as β^+^ or β^–^ has also been demonstrated in succession [6]. Presently, the studies of *in vivo* CLI in small living animals mainly focus on 2D imaging [4]–[13]. However, the 2D images cannot provide the depth and quantitative information of the radionuclide tracer. Li *et al*. presented 3D Cerenkov luminescence tomography (CLT) based on a homogeneous mouse model using multiple views [14]. Multiple views could reduce the ill-posedness of the inverse problem and improve the reconstruction equality of single-spectral CLT [14]. However, the acquisition of multiple views increases the complexity of the experiment. And the hypothesis of a homogeneous optical parameter will lead to inaccurate source reconstruction results [17], [18]. In our previous study, we performed *in vivo* 3D CLT based on a heterogeneous mouse model with an implanted Na^131^I radioactive source [15]. Our results showed that the reconstruction based on a heterogeneous mouse model was more accurate in localization than using the homogeneous one [15]. Nevertheless, the reconstruction results were not very encouraging based on a single spectrum 675–775 nm because CLT is a very challenging ill-posed inverse problem. Spinelli *et al*. described a multispectral diffuse CLT (msCLT), which was based on a set of 2D planar images acquired using a number of narrow bandpass filters [16]. Multispectral data could effectively improve the ill-posedness of the inverse problem; however an increase in the known data also reduces the reconstruction efficiency [19]. On the other hand, compared with the other optical molecular imaging, the *in vivo* Cerenkov luminescent signal is weak when the radiopharmaceutical is at a safe injection dose. Hence, the detected multispectral data will be weaker after using more than one filter, which will increase the difficulty of the source reconstruction. Furthermore, the use of a number of filters to obtain 2D planar images for 3D reconstruction costs more acquisition time.

In this paper, we presented a semi-quantitative Cerenkov radiation spectral characteristic-based Cerenkov luminescent source reconstruction method called hybrid spectral CLT, which was based on a single 2D planar image. It was unnecessary to use filters to acquire luminescent images, yet the information about wavelengths was incorporated in the reconstruction process, thus more encouraging results were obtained. We constructed the implantation mouse model implanted with a 400 *µ*Ci Na^131^I radioactive source and the physiological mouse model received an intravenous tail injection of 400 *µ*Ci radiopharmaceutical Iodine-131 (I-131) to validate the performance of the hybrid spectral CLT and compared the reconstruction results, acquisition and image reconstruction time with that of single-spectral and multispectral CLT. Results showed that the reconstruction based on the hybrid spectral CLT was more accurate in localization and quantification than using single-spectral CLT, and was more efficient in the *in vivo* experiment compared with multispectral CLT. Based on the hybrid spectral reconstruction method, we performed 3D noninvasive, longitudinal observations of I-131 uptake in the thyroid and quantified I-131 uptake *in vivo* by means of hybrid spectral CLT/CT. The results were consistent with SPECT imaging and the *ex vivo* biodistribution experiment further verified the results of hybrid spectral CLT.

## Results

### Spectral Characteristics of the Na^131^I Radioactive Source

**Figure 1:**
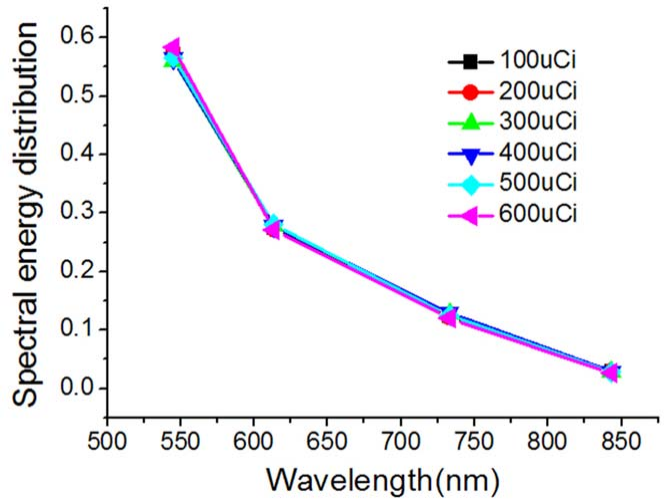
The measured spectra of the emitted luminescence from the Na^131^I radioactive sources. Na^131^I radioactive sources (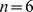) were made of glass vessels filled with Na^131^I and were acquired for luminescent images by using the standard filter set installed on the IVIS system including 515–575 nm (GFP), 575–650 nm (DsRED), 695–770 nm (Cy5.5) and 810–875 nm (ICG). The activities of Na^131^I were 100 *µ*Ci, 200 *µ*Ci, 300 *µ*Ci, 400 *µ*Ci, 500 *µ*Ci and 600 *µ*Ci respectively. The percentages of optical signal intensity at four different discrete spectral ranges were approximately 0.572, 0.276, 0.124, and 0.028 respectively. Spectral energy distribution of the emitted light from the Na^131^I radioactive source with various activities is shown in Fig. 1.

To perform hybrid spectral CLT, the spectral characteristics of the Na^131^I radioactive source with various activities were investigated. The measured spectra of the emitted luminescence from the Na^131^I radioactive sources presented a broad distribution from 500 nm to 900 nm, and the optical signals emitted from the Na^131^I radioactive sources decreased with increasing wavelength (Fig. 1), which was consistent with our previous findings [15]. The spectrum presented a 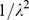
dependence [3], [4]. We found that the percentages of optical signal intensity at four different discrete spectrum ranges including 515–575 nm, 575–650 nm, 695–770 nm and 810–875 nm were approximately 0.572, 0.276, 0.124, and 0.028 respectively. They were used in hybrid spectral CLT. Furthermore, the proportions were irrelevant to the activity of the Na^131^I radioactive sources. Hence, we can reconstruct Na^131^I with various activities using hybrid spectral CLT.

### Hybrid Spectral CLT of the Implantation Mouse Model

**Figure 2:**
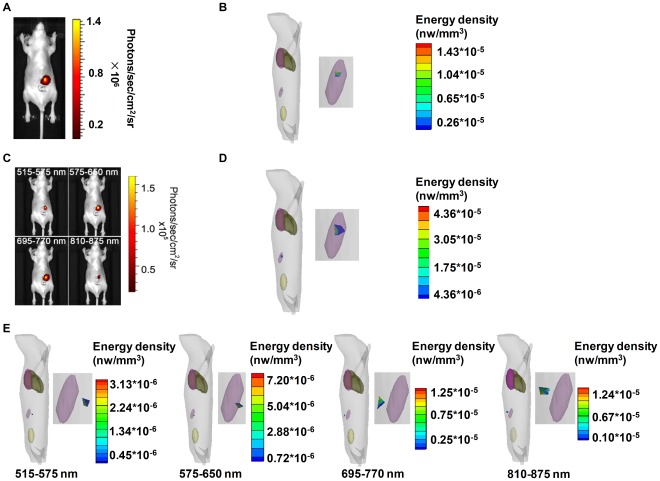
Hybrid spectral, multispectral and single-spectral CLT of a mouse implanted with a Na^131^I radioactive source. Fig. 2(A) is the single luminescent image of the athymic nude mouse implanted with the 400 *µ*Ci Na^131^I radioactive source without using filters, which is in pseudo-color superimposed on the corresponding photograph. Fig. 2(B) shows the 3D rendering of the reconstruction results of hybrid spectral CLT based on the single luminescent image. Fig. 2(C) consists of multispectral measurements of the mouse with the implanted 400 *µ*Ci Na^131^I radioactive source acquired by a set of four filters including 515–575 nm, 575–650 nm, 695–770 nm and 810–875 nm filters respectively. Fig. 2(D) has the 3D rendering of the reconstruction results based on the multispectral measurements. The 3D renderings of the reconstructed source distribution based on the four single spectra as shown in Fig. 2(E), including 515–575 nm, 575–650 nm, 695–770 nm and 810–875 nm respectively.

The luminescent image of the mouse implanted with the 400 *µ*Ci Na^131^I radioactive source was acquired without using filters, which is in pseudo-color superimposed on the corresponding photograph (Fig. 2A). We observed optical signals in the abdomen of the mouse. The absorption coefficient and reducing scattering coefficient of the mouse biological tissues are calculated and listed in Table 1. Using the previously described semi-quantitative Cerenkov radiation spectral characteristic-based source reconstruction method, the 3D distribution of the internal implanted Na^131^I radioactive source was reconstructed and shown in 3D rendering (Fig. 2B). In order to analyze the results quantitatively, we defined the distance error 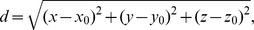 where 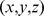 is the coordinate of the reconstructed source with maximum density, and 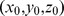 is that of the actual source. The reconstructed energy is defined as 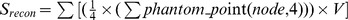, where the 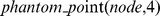 is the density of the tetrahedral nodes, and 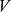 is the volume of the tetrahedron. The activity of the source can be calculated through the linear relationship between the reconstructed energy and the source’s activity (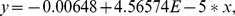 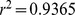) (as shown in Figure S1). The purple object similar to a cylinder was the implanted radioactive source and the coordinates could be obtained via micro-CT images as (115.84, 107.24, 30.00) mm. The reconstructed source was a cluster of tetrahedrons because of the tetrahedral mesh generation. The reconstructed sources are clearly magnified and shown in Fig. 2. The location of the reconstructed source was (115.79, 107.57, 30.59) mm, the distance error was 0.68 mm and the reconstructed source energy was 1.11×10^−2^ nw. The reconstructed source activity was 386 *µ*Ci and the activity error was 3.51% (Table 2).

**Table 1:** Optical parameters for the mouse organ regions.

Coefficient	Adipose	Heart	Lungs	Liver	Stomach	Kidneys	Bone	Spleen	Bladder	Intestines
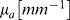	0.1017	1.5477	4.6832	9.2860	0.3082	1.7334	1.5233	9.2860	0.1017	0.2891
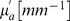	1.2929	1.1674	2.3271	0.7786	1.6320	2.7599	3.0393	0.7786	1.2929	1.3548

### Single-spectral CLT and Multispectral CLT of the Implantation Mouse Model

To compare the reconstruction results of hybrid spectral CLT with that of single-spectral CLT and multispectral CLT, we reconstructed the 3D distribution of the internal implanted radioactive source based on the single spectrum and multispectral measurement data respectively.

The four luminescent views in pseudo-color superimposed on the corresponding photographs of the same mouse were acquired from a set of four filters installed on the IVIS system (Fig. 2C). The maximum optical signal was observed when the 695–770 nm filter was used. The second was observed for the 575–650 nm filter, the third for the 515–575 nm filter, and the optical signal was the lowest when the 810–875 nm filter was used. The reconstruction results based on the multispectral measurements were shown in 3D rendering (Fig. 2D). The location of the reconstructed source was (115.38, 107.57, 29.82) mm, the distance error was 0.59 mm, the reconstructed source energy was 1.13×10^−2^ nw, the reconstructed source activity was 390 *µ*Ci and the activity error was 2.46% (Table 2).

**Table 2:** The reconstruction results for the implantation mouse model based on hybrid spectral CLT, single-spectral CLT and multispectral CLT.

Spectrum	Actual sourcecenter (mm)	Reconstructed sourcelocation (mm)	Locateddeviation (mm)	Reconstructedenergy (×10^−5 ^nw)	Activityerrors %
Hybrid spectral	(115.84, 107.24, 30.00)	(115.79, 107.57, 30.59)	0.68	1114.30	3.51
515–575 nm	(115.84, 107.24, 30.00)	(115.24, 109.89, 28.20)	3.26	3.15	179.60
575–650 nm	(115.84, 107.24, 30.00)	(116.12, 108.43, 27.93)	2.40	6.85	178.03
695–770 nm	(115.84, 107.24, 30.00)	(118.77, 105.53, 30.05)	3.40	101.72	142.95
810–875 nm	(115.84, 107.24, 30.00)	(115.05, 105.18, 29.37)	2.30	89.81	146.85
Multispectral	(115.84, 107.24, 30.00)	(115.38, 107.57, 29.82)	0.59	1134.53	2.4564

The reconstruction results based on the above four single spectra were shown in 3D rendering (Fig. 2E). The distance error of single-spectral CLT was 2.84±0.57 mm. The located deviation based on the 810–875 nm spectrum was 2.30 mm, which was the lowest amount for all of the reconstruction results. All of the reconstructed energy based on the four single spectra was very inaccurate and the activity error was 161.86±0.20% (Table 2). It was obvious that the reconstruction results in localization and quantification based on the multispectral measurements were more accurate than those based on single spectral data.

Through the comparisons of the reconstruction results of the hybrid spectral CLT and that of single-spectral CLT, these data demonstrated the results of hybrid spectral CLT which were more accurate in the reconstructed location and quantification than that of single-spectral CLT. Compared with multispectral CLT, the distance error of hybrid spectral CLT was a little larger than that of multispectral CLT, and it was a little less accurate in quantification than multispectral CLT. Furthermore, we compared the acquisition and image reconstruction time with these methods, and the results are shown in Table 3. There was not a large difference in both the acquisition and image reconstruction time between hybrid spectral CLT and single-spectral CLT. For multispectral CLT, its acquisition time was four times as that of hybrid spectral CLT, and image reconstruction time was about 41 times slower than hybrid spectral CLT. Integrating the reconstruction results and required time, the hybrid spectral CLT showed better performance than the two other methods.

### Hybrid Spectral CLT of I-131 Uptake in the Bladder of the Physiological Mouse Model

**Figure 3:**
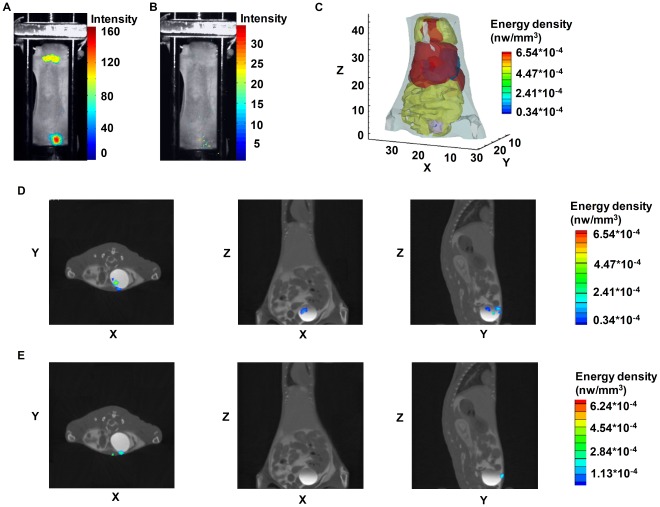
The biodistribution of I-131 uptake in the mouse bladder of hybrid spectral CLT. An athymic nude mouse received the intravenous tail injection of 400 *µ*Ci I-131. Fig. 3(A) is the single luminescent image of the mouse 2 h after the injection which was acquired without using filters; this is depicted in pseudo-color superimposed on the corresponding photograph. The luminescent image of the mouse using the 700–40 nm filter is shown in Fig. 3(B) where there is almost no optical signal when using the other filters including 580–40 nm, 620–40 nm, and 660–40 nm. Fig. 3(C) is the 3D radiopharmaceutical I-131 biodistribution rendering of hybrid spectral CLT. Fig. 3(D) and (E) are the reconstruction results in horizontal, coronal, and sagittal views of the hybrid spectral CLT and the single-spectral CLT based on the 700–40 nm spectrum.

We observed optical signals in the neck and abdomen of the mouse two hours after the intravenous tail injection with 400 *µ*Ci I-131 (Fig. 3A). To confirm the performance of hybrid spectral CLT in a realistic and more biologically representative mouse model, we also acquired multispectral luminescent images of the mouse using four filters, including 580–40 nm, 620–40 nm, 660–40 nm, and 700–40 nm. A very weak optical signal was obtained using the 700–40 nm filter, and almost no signal was acquired by using the other filters (Fig. 3B).

We then performed the 3D reconstruction of I-131 uptake in the mouse bladder using hybrid spectral CLT. Optical parameters of biological tissues used in the reconstruction are listed in Table 1. Reconstructed I-131 was clearly distributed in the mouse bladder in the 3D radiopharmaceutical I-131 biodistribution rendering of the heterogeneous mouse model (Fig. 3C). The geometrical center of the bladder was defined as the actual source location, which could be obtained by micro-CT images at (18.24, 25.76, 3.68) mm. The location of the reconstructed source was (18.86, 27.04, 3.54) mm, the distance error between the actual source and the reconstructed one was 1.43 mm, the reconstructed total energy of I-131 was 1.34×10^−4^ nw, and the reconstructed source activity was 145 *µ*Ci (Table 4). Nuclear signals appeared in the mouse bladder in SPECT imaging (data not shown), which was approximately consistent with that of hybrid spectral CLT.

**Table 3:** Comparisons of hybrid spectral CLT, single-spectral CLT and multispectral CLT based on the acquisition and image reconstruction time.

	Acquisition time (min)	Image reconstruction time (min)
Hybrid spectral	5	5.24
695–770 nm	5	4.87
575–650 nm	5	4.74
515–575 nm	5	4.72
810–875 nm	5	4.92
Multispectral	20	218.12

**Table 4:** The reconstruction results of I-131 uptake in the mouse bladder using hybrid spectral CLT and single-spectral CLT.

	Actual sourcecenter (mm)	Reconstructed source location (mm)	Locateddeviation (mm)	Reconstructedenergy (×10^−5 ^nw)	Activity(*µ*Ci)
Hybrid spectral CLT	(18.24, 25.76, 3.68)	(18.86, 27.04, 3.54)	1.43	13.43	145.31
Single-spectral CLT	(18.24, 25.76, 3.68)	(20.40, 29.86, 3.77)	4.64	102.12	164.73

The reconstruction results of hybrid spectral CLT and single-spectral CLT based on the 700–40 nm spectrum were in horizontal, coronal, and sagittal views across the geometric center of the bladder respectively (Fig. 3D and E). It was clear that the reconstructed location of hybrid spectral CLT was more accurate than that of single-spectral CLT. For single-spectral CLT, the distance error between the actual source and the reconstructed one was 4.64 mm, the total energy of the reconstructed source was 1.02×10^−3^ nw, and the reconstructed source activity was 164 *µ*Ci (Table 4). Results showed that hybrid spectral CLT could accurately reconstruct the I-131 uptake in the mouse bladder and was more applicable than multispectral CLT in the *in vivo* experiment.

### 3D Noninvasive Longitudinal Observation of Radiopharmaceutical I-131 Uptake in the Thyroid and the Quantification of I-131 Uptake in vivo using Small Animal Hybrid Spectral CLT/CT

**Figure 4:**
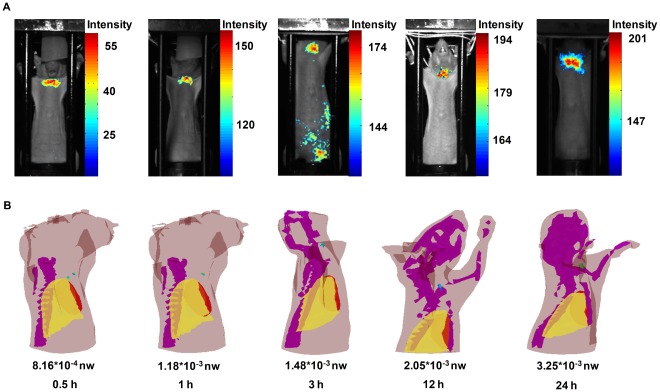
Noninvasive longitudinal observations of I-131 uptake in the thyroid using CLI and 3D CLT. Fig. 4(A) shows the 2D luminescent images of I-131 uptake in the athymic nude mouse thyroid at 0.5 h, 1 h, 3 h, 12 h, and 24 h after the intravenous tail injections of 450 *µ*Ci of I-131. Fig. 4(B) portrays 3D noninvasive longitudinal observations of I-131 uptake in the thyroid and the quantification of I-131 uptake *in vivo* using small animal hybrid spectral CLT/CT.

**Figure 5:**
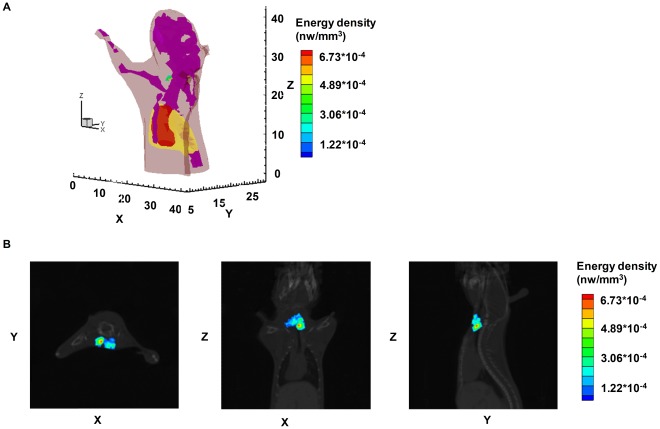
The CLT of athymic nude mice at 24 h after injection. The athymic nude mouse received the intravenous tail injection of 450 *µ*Ci I-131. Fig. 5(A) is the 3D rendering of the I-131 uptake in the thyroid using hybrid spectral CLT at 24 h post-injection. Fig. 5(B) has the reconstruction results of hybrid spectral CLT of I-131 uptake in the thyroid at 24 h after the injection in horizontal, coronal, and sagittal views respectively.

We observed optical signals in the mouse neck from the 2D planar luminescent images obtained at 0.5 h, 1 h, 3 h, 12 h, and 24 h after the intravenous tail injections of 450 *µ*Ci I-131 (Fig. 4A). We performed a 3D noninvasive longitudinal observation of I-131 uptake in the thyroid and the quantification of I-131 uptake *in vivo* by means of hybrid spectral CLT (Fig. 4B). The reconstructed energy reflected the I-131 uptake in the thyroid. I-131 uptake increased with the collection time point and its maximal energy appeared at 24 h (Table 5). The 3D distribution of I-131 at 24 h after the injection using hybrid spectral CLT is shown in Fig. 5A. The reconstruction results in horizontal, coronal, and sagittal views show the I-131 uptake in the thyroid (Fig. 5B). Nuclear signals were observed in the mouse neck and there was no signal in the other organs at 24 h after the injection (data not shown), which was similar to hybrid spectral CLT.

**Table 5:** The reconstructed energy and activity of I-131 uptake in the thyroid at collection time points.

Collection time points (h)	Reconstructed energy (×10^−5 ^nw)	Activity (*µ*Ci)
0.5	81.628	160.24
1	117.51	168.10
3	147.92	174.76
12	204.6	187.18
24	324.65	213.47

**Figure 6:**
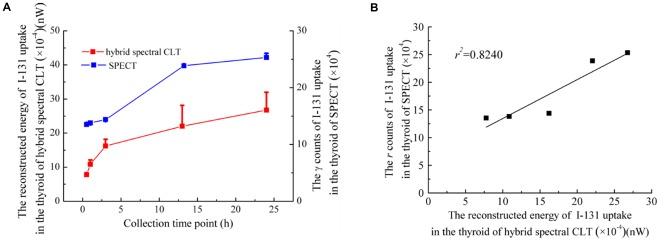
The correlation analysis between hybrid spectral CLT and SPECT. The reconstructed energy of I-131 uptake in the thyroid as a function of the collection time point is described in Fig. 6(A) (red line). The gamma ray counts of I-131 uptake for SPECT imaging as a function of the collection time point is depicted in Fig. 6(A) (blue line). There was a robust correlation between the reconstructed energy of I-131 versus the gamma ray counts of I-131 (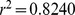) as shown in Fig. 6(B).

The reconstructed energy of the I-131 uptake in the thyroid increased with increasing collection time points and maximal energy appeared at 24 h (Fig. 6A (red line)). The gamma ray counts of the I-131 uptake in the thyroid increased with increasing collection time points (Fig. 6A (blue line)). There was a robust correlation between the reconstructed energy of I-131 versus the gamma ray counts of I-131 (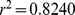) (Fig. 6B). This indicated the capacity of hybrid spectral CLT for quantifying the I-131 uptake in the thyroid compared with SPECT imaging.

**Figure 7:**
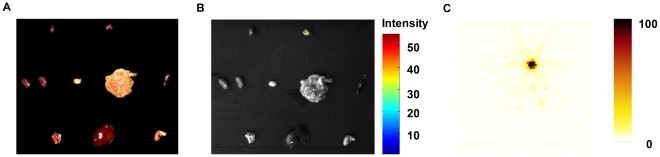
The *ex vivo* biodistribution experiment of the mouse. The mice received euthanasia and dissection at 24 h after the intravenous tail injections of 450 *µ*Ci I-131. Fig. 7(A) and (B) are the photographs and luminescent images of the mouse organs, including the heart, lungs, liver, stomach, kidneys, bladder, spleen, intestines, and thyroid respectively. The optical signals were observed in the mouse thyroid and there were no signals in the other mouse organs (Fig. 7(B)). Fig. 7(c) is the nuclear imaging of the mouse organs. The thyroid was clearly imaged but other mouse organs were not.

The mouse was dissected at 24 h after the intravenous tail injection of 450 *µ*Ci I-131, and the mouse organs included the heart, lungs, liver, stomach, kidneys, bladder, spleen, intestines, and thyroid (Fig. 7A). The optical signals were observed in the mouse thyroid and there were no signals in the other mouse organs (Fig. 7B). It stated clearly that the radiopharmaceutical I-131 biodistribution occurred in small living animals. Fig. 7C is the nuclear imaging of the mouse organs. The thyroid was clearly imaged but other mouse organs were not. The results of CLI of the mouse organs were concurrent with that of SPECT imaging. The *ex vivo* biodistribution experiment further confirmed the I-131 uptake in the thyroid of hybrid spectral CLT.

Our data indicated that hybrid spectral CLT combined with small animal CT can be used for 3D noninvasive longitudinal monitoring of I-131 uptake in the thyroid and the quantification of I-131 uptake *in vivo*.

## Discussion

Multispectral imaging can reduce the ill-posedness of the inverse problem in CLT and significantly improve the reconstruction quality. An increase in the known data also reduces the reconstruction efficiency because the dimension of the Jacobian matrix becomes very large for most iteration-based algorithms [19]. On the other hand, the Cerenkov luminescent signal is very weak when the radiopharmaceutical has a low radiation dose. Hence, it is difficult to obtain multispectral data using filters. Furthermore, the use of a number of filters to obtain 2D planar images for 3D reconstruction requires more acquisition time. In this paper, we present a semi-quantitative Cerenkov radiation spectral characteristic-based source reconstruction method for CLT known as hybrid spectral CLT, which is based on a single 2D planar image acquired without using filters. The reconstruction results of the implanted Na^131^I radioactive source demonstrated greater accuracy in location and quantification of hybrid spectral CLT than for single-spectral CLT. Compared with multispectral CLT, the distance error of the hybrid spectral CLT was 0.68 mm, which was slightly larger than for multispectral CLT, and it was a little less accurate in quantification than multispectral CLT. However, the results were encouraging. The acquisitions and image reconstruction time can be greatly shortened simultaneously. In addition, the biodistribution results of I-131 uptake in the mouse bladder showed the ability of hybrid spectral CLT to locate the position correctly and the superiority for the reconstruction of the weak Cerenkov luminescent signal compared with multispectral CLT. Hybrid spectral CLT does not need to use filters for acquiring luminescent images; however, the information about wavelengths was incorporated into the reconstruction process. Hence, more encouraging results were obtained without increasing acquisition time.

The spectral distribution characteristics of the Na^131^I radioactive source with various activities were investigated, which was important for the Cerenkov source reconstruction in hybrid spectral CLT. Experimental results suggested that the percentages of optical signal intensity at the four different discrete spectrum ranges were irrelevant to the activity of the Na^131^I radioactive sources. Thus, we can reconstruct the radionuclide tracer with various activities of the hybrid spectral CLT. Additionally, we speculated that the percentages possibly had no relevance with the variety of radionuclide tracers according to the spectral distribution of various radionuclide tracers [6]. Consequently, it was very convenient to reconstruct the 3D biodistribution of various radionuclide tracers in small living animals using the hybrid spectral CLT without computing the optical parameters during the reconstruction process and using any filters for acquiring images.

The choice of implanting an 131-I source in an animal is not at all physiological, so in order to verify the performance of hybrid spectral CLT in a real *in vivo* experiment, we performed a much easier, but at the same time more elegant, realistic and more biologically oriented solution for imaging I-131 uptake in the mouse bladder. Based on hybrid spectral CLT, the located deviation of the I-131 uptake in the mouse bladder was 1.43 mm. For single-spectral CLT, the distance error was 4.64 mm. Experimental results suggested that hybrid spectral CLT could accurately reconstruct I-131 uptake in the mouse bladder. Here, we could not perform multispectral CLT due to the non-acquisition of multispectral measurements. It showed that hybrid spectral CLT was more applicable than multispectral CLT in the *in vivo* experiment.

The located deviation of the implanted radioactive source was 0.68 mm, and the located deviation of the I-131 uptake in the mouse bladder was 1.43 mm, which was worse than in the implantation mouse model. It was probably due to the use of the implanted point-like Cerenkov source which assisted in the convergence of the reconstruction algorithm.

As we all know, the thyroid gland situated in the anterior neck below the skin and muscle layers is the biggest gland in the neck. It takes the shape of a butterfly with the two wings being represented by the left and right thyroid lobes. The function of the thyroid is to regulate the body’s metabolism. In clinics, the thyroid scan is employed to determine the position, shape and size of the thyroid gland and the thyroid uptake is used to estimate the function of the gland. Iodine concentrates in the thyroid. This is the basis for morphological and functional imaging. The physical half-life of I-131 radiotracer is 8.04 day, the maximal energy of beta particles is 0.606 MeV, and the energy of γ rays is 0.364 MeV. It satisfies the threshold energy for the production of Cerenkov radiation in tissues [4]. Based on Cerenkov radiation, we performed a 3D noninvasive longitudinal observation of I-131 uptake in the thyroid by means of hybrid spectral CLT/CT. The 3D visualization of longitudinal observations clearly showed that there was I-131 uptake in the thyroid and that the reconstructed energy of I-131 uptake in the thyroid increases with acquisition time. The reconstructed location of I-131 was not verified because the mouse thyroid was similar to connective tissue, meaning that CT could not distinguish it from the surrounding tissues. Nevertheless, it was roughly affirmed by SPECT images. Furthermore, in the *ex vivo* biodistribution experiment, the optical signals were observed in the mouse thyroid and there were no signals in the other mouse organs, which was concurrent with the nuclear images (Fig. 7B). It further confirmed I-131 uptake in the thyroid for hybrid spectral CLI.

We quantified radiopharmaceutical I-131 uptake in the thyroid *in vivo* using hybrid spectral CLT/CT. There was a robust correlation between the reconstructed energy of I-131 versus the gamma ray counts of I-131 (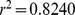) (Fig. 6B). Our study indicated the potential of hybrid spectral CLT for clinical application in the future, such as determining thyroid size, function, and position, quantitative thyroid uptake, treatment of hyperthyroidism and cancer, and detection of thyroid metastases and assessment of response to therapy. Compared with nuclear imaging, it has more advantages, such as having a lower cost of using an optical instrument, high throughput, and high specificity. Therefore, we anticipate that CLT could be used in clinics in the future.

Normally, our imaging object is a mouse model, but for bigger imaging objects, such as the size of larger animals or humans, our proposed algorithm would encounter some difficulties. Firstly, due to the intrinsic spectral and intensity limitation of the Cerenkov luminescence signal in the animal model, which is unable to penetrate the thick biological tissue and be detected with a highly sensitive charged coupled device. Furthermore, the existing optical coefficients in this manuscript were calculated according to the literature [18]. We could not find more comprehensive optical coefficients for large animals, which will induce great difficulties for heterogeneous reconstruction. Additionally, the acquisition of large animals will bring a large amount of data. Limited by the current computer hardware technology, the inversion of a large matrix is a difficult problem for source reconstruction.

In order to provide a comparison with conventional SPECT imaging, we used a SPECT-CT system with poor spatial resolution. This is a suboptimal imaging procedure for small animal imaging. However, the most important point to make here is that fused SPECT-CT images were used to roughly compare them with reconstructed Cerenkov images. In later research, we will consider developing an animal multimodality CLT/SPECT/CT system to perform the biomedical applications.

In this paper, we revisited the problem of reconstructing the 3D radiopharmaceutical biodistribution in small living animals in CLT. We have presented a modified reconstruction method based on Cerenkov radiation spectral characteristics to efficiently reconstruct the biodistribution of the radionuclide tracer. It does not have to use filters for acquiring luminescent images for reconstruction; however, the information about wavelengths was incorporated into the reconstruction process. Hence, the more encouraging results were obtained without increasing acquisition time. We have experimented on an implantation mouse model and a physiological mouse model and compared our method and results with single-spectral and multispectral CLT. Furthermore, we performed 3D noninvasive longitudinal monitoring of I-131 uptake in the thyroid and quantified I-131 uptake *in vivo* by means of small animal hybrid spectral CLT/CT. Our study showed that our approach was efficient in reconstructing the biodistribution of the radionuclide tracer, and 3D visualization of I-131 uptake in the thyroid suggested a linear relationship between the reconstructed energy of CLT and the gamma ray counts for SPECT which could provide more quantitative information. We believe that our approach delivers valuable information and provides an excellent tool that facilitates more detailed exploration for CLT clinical application.

## Materials and Methods

All animal procedures were in accordance with the Fourth Military Medical University (FMMU) approved animal protocol.

### Preparation of Radioactive Sources

Na^131^I radioactive sources (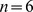) were made of glass vessels filled with Na^131^I. The activities were 100 *µ*Ci, 200 *µ*Ci, 300 *µ*Ci, 400 *µ*Ci, 500 *µ*Ci and 600 *µ*Ci respectively. The volume of the Na^131^I radioactive source was about 13 mm^3^ and the solvent of the radioactive source was normal saline. The dimensions of the columniform glass vessel were approximately 3.42 mm in diameter and 7 mm in length. A control source was filled with a nonradioactive NaI solution.

### Establishment of the Implantation Mouse Models and the Physiological Mouse Models

The implantation mouse models were established by implanting a 400 *µ*Ci Na^131^I radioactive source into an athymic nude mouse belly (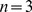, weight 16±3 g). The mice underwent aseptic celiotomy and the depth of the embedded radioactive source was approximately 4 mm from the top surface of the mouse. The athymic nude mice (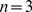, weight 17±3 g) received an intravenous tail injection of 400 *µ*Ci I-131 to construct the physiological mouse models. The athymic nude mice (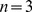, weight 22±2 g) received intravenous tail injections of 450 *µ*Ci I-131 and were used for the 3D noninvasive, longitudinal observations of I-131 uptake in the thyroid using hybrid spectral CLT. In the experiment, the mice were anesthetized using isoflurane (2%).

### Cerenkov Luminescence Imaging and CT Imaging

To investigate the spectral characteristic of the emitted light from the Na^131^I radioactive sources with various activities, the previously described Na^131^I radioactive sources (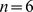) were imaged for acquiring optical signals with the Xenogen *In Vivo* Imaging System (IVIS Kinetic, Caliper Life Sciences) using four filters installed on the IVIS system including 515–575 nm (GFP), 575–650 nm (DsRED), 695–770 nm (Cy5.5) and 810–875 nm (ICG). Regions of interest (ROIs) of the same area were drawn over the optical images of the six radioactive sources, and the average radiances were read by Living Image 3.2 software (IVIS Kinetic, Caliper Life Sciences), which provided the intensities of the optical signals.

To reconstruct the implanted 400 *µ*Ci Na^131^I radioactive source using hybrid spectral CLT, the mice (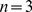) with the implanted 400 *µ*Ci Na^131^I radioactive source were acquired for a single luminescent image by the IVIS system without using any filters. Comparing single-spectral CLT and multispectral CLT, the mice were also acquired for multispectral images using the previously described four filters. All luminescent images were acquired with a binning value of 4, integration time of 5 min and aperture number *f_num_* = 1.

To reconstruct the I-131 uptake in the bladder, the athymic nude mice (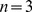) were affixed in an animal-imaging holder in a vertical position two hours later after the intravenous tail injection of 400 *µ*Ci I-131 and imaged for a single luminescent image of the mouse abdomen with a dual-modality ZKKS-Direct3D molecular imaging system (jointly developed by Guangzhou Zhongke Kaisheng Medical Technology CO., Ltd, Xidian University and Institute of Automation, CAS). To compare the results of the hybrid spectral CLT with single-spectral and multispectral CLT, the mice were then imaged using a number of filters including 580–40 nm, 620–40 nm, 660–40 nm, and 700–40 nm.

For performing 3D longitudinal observations of I-131 uptake in the thyroid, athymic nude mice (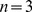) were acquired for luminescent images of the mouse neck at 0.5 h, 1 h, 3 h, 12 h, and 24 h after the intravenous tail injections of 450 *µ*Ci I-131 using the dual-modality ZKKS-Direct3D molecular imaging system. To further confirm the I-131 uptake in the thyroid through the *ex vivo* experiment, the mice were euthanized and dissected at 24 h after the intravenous tail injections of 450 *µ*Ci I-131. The mouse organs, including the heart, lungs, liver, stomach, kidneys, bladder, spleen, intestines, and thyroid were acquired for luminescent images. All luminescent images were acquired with a binning value of 4, integration time of 5 min and aperture number *f_num_* = 2.8.

In order to perform the 3D reconstruction of the radiopharmaceutical biodistribution in the living mouse, both the athymic nude mice who received intravenous tail injections of 400 *µ*Ci of I-131 and the athymic nude mice who received intravenous tail injections of 450 *µ*Ci of I-131 were scanned for acquiring structural information by the dual-modality ZKKS-Direct3D molecular imaging system in the same vertical position as the optical imaging immediately after celiac administration of the contrast material diatrizoate meglumine injection using the following imaging parameters: 50 kV and 1 mA. To accelerate the speed of 3D computed tomography reconstruction, the Feldkamp-Davis-Kress (FDK) algorithm on the commodity GPU using an acceleration scheme was employed for the 3D reconstruction of CT images [20]. The algorithm performed the reconstruction on a 256×256×256 volume (160 *µ*m×160 *µ*m×160 *µ*m voxel size).

### SPECT/CT Imaging

To compare CLT images with SPECT images, the athymic nude mice (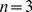) with the intravenous tail injections of 400 *µ*Ci I-131 immediately underwent SPECT imaging after CLT. In the longitudinal observation experiment of I-131 uptake in the thyroid, the athymic nude mice (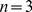) were immediately acquired for nuclear images after CLT at 0.5 h, 1 h, 3 h, 12 h, and 24 h after the intravenous tail injections of 450 *µ*Ci I-131. SPECT imaging was performed with the SPECT-CT system (Symbia T2, Siemens) using the following imaging parameters: 64 frames, 64×64 pixels and zoom = 1. To perform the correlation analysis between the hybrid spectral CLT and SPECT, the planar SPECT images were acquired with the parameters: 128×128 pixels and zoom = 1. ROIs were drawn over the nuclear images to acquire 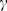 counts. In the experiment, the mice were affixed on the hardboard in the supine position using adhesive tape. For acquiring structural information, the mice were scanned with the CT system after the intravenous tail injections with contrast media.

### The Semi-quantitative Cerenkov Radiation Spectral Characteristic-based Cerenkov Luminescent Source Reconstruction Method

The propagation of Cerenkov photons in biological tissues can be described by the steady-state diffusion equation (DE) and Robin boundary condition as follows [21]–[25]:
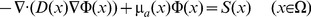
(1)

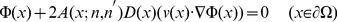
(2)


The related parameters are detailed in Ref. [21]–[25].

Based on the finite element theory, a linear relationship between the Cerenkov luminescent source and the measured Cerenkov signal on the body surface can be described as follows:
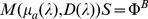
(3)where 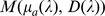 is the system matrix which is related to the tissue optical properties; 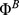 represents the nodal flux density at the boundary.

Presently, both single spectral and multispectral methods were employed for the solution of 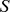. In single-spectral CLT, the equation can be expressed as follows:
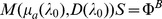
(4)


The optical parameters are usually calculated based on 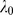, which is the center wavelength of the band pass filter used in data acquisition.

In multispectral CLT, equation (3) can be expressed as follows:
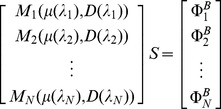
(5)where 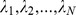 are the center wavelengths of a number of band pass filters which are used in the multispectral data acquisition; 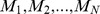 represent the system matrix of the different spectra 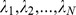 respectively; and 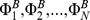 denote the nodal flux density at the boundary of different spectra 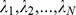.

In single-spectral CLT, the optical parameters are calculated according to the certain wavelength of the band pass filter, which cannot accurately describe the photon propagation in biological tissues. Additionally, it is difficult to acquire a weak Cerenkov luminescence signal using a filter. For multispectral CLT, the multispectral data could effectively improve the ill-posedness of the inverse problem because the acquisition of multispectral information increases the amount of known boundary measurements. However, the increase of computational cost significantly reduces the reconstruction efficiency. More importantly, since the emitted light from the Cerenkov luminescent source is quite weak, the usage of a number of filters may increase the difficulty to obtain the multispectral measurements. Furthermore, the acquisition of multispectral data using a number of filters requires greater acquisition time.

On the basis of the above analysis, we presented a modified CLT named hybrid spectral CLT to reconstruct the biodistribution of the radionuclide tracer. In our method, data in the entire spectrum of the Cerenkov signals were employed in the reconstruction of the Cerenkov source. The optical parameters of the tissues over the entire spectrum were estimated based on the spectral characteristics of the Cerenkov source and could be determined as follows:
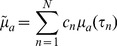
(6)

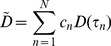
(7)where 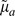 is the estimated absorption coefficient in the entire spectrum of Cerenkov light; 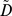 is the related diffusion coefficient; 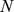 is the number of spectra for reconstruction; here, 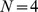; 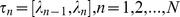; and 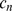 is the percentage of optical signal intensity at 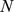 different discrete spectral ranges and can be calculated by the measured spectra of the emitted luminescence from the radionuclide tracer.

Thus, Eq. (3) can be converted into the following formula in our proposed method:
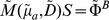
(8)where 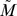 is the system matrix based on the hybrid spectral measurements; 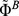 is the measured Cerenkov light without any filters.

Applying the adaptive *hp* strategy [26], Eq. (8) can be transformed to the following equation:
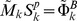
(9)where 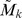 is the system matrix for the 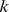-th level mesh which is related to the estimated optical properties; 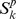 is the Cerenkov light source distribution located in the permissible source region that is determined by a priori knowledge; and 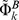 is the nodal flux density on the surface. In the mouse experiments, the surface nodal flux density was obtained by mapping the measured 2D Cerenkov images onto the 3D body surface [27].

Because of the ill-posed nature of CLT, it is difficult to solve Eq. (9) directly. The classical Tikhonov regularization method can be used to solve Eq. (9). The following optimization problem is defined to determine the Cerenkov light source distribution:
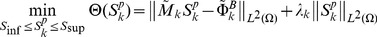
(10)where 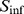 and 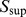 are the lower and upper bounds of the Cerenkov light source power density and 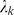 denotes the regularization parameter. 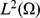 is the weight matrix and satisfies 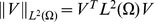. This minimization problem is solved by a modified Newton method with an active set strategy [28].

To quantify radiopharmaceutical uptake *in vivo* in a small animal by means of hybrid spectral CLT/CT, a CCD camera was calibrated using an integrating sphere of 12 inches in diameter (USS-1200V-LL Low-Light Uniform Source, Labsphere, North Sutton, NH). The calibrated method was introduced in the literature [29]. The calibration formula is given as follows:
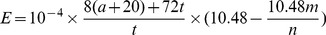
(11)where 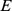 (nW/mm^2^) is the irradiance intensity on the mouse surface; 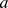 is the pixel gray value of the Cerenkov luminescent image; 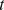 (s) is the exposure time for the Cerenkov luminescent image acquisition; 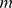 (mm) is the distance between the center of the lens front face and the intersection of the principal optical axis and the mouse frontal surface; and 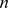 (mm) is the distance between the edge of the cylindrical lens and the intersection of the principal optical axis and the mouse frontal surface.

## Supporting Information

Figure S1
**The correlation between the reconstructed source energy and activity.** Athymic nude mice (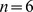, weight 16±2 g) underwent aseptic celiotomy and were implanted with 100 *µ*Ci, 200 *µ*Ci, 300 *µ*Ci, 400 *µ*Ci, 500 *µ*Ci and 600 *µ*Ci Na^131^I radioactive sources into the mouse bellies respectively. The depth of the embedded radioactive source was approximately 4 mm from the top surface of the mouse. We then acquired a single luminescent image with the IVIS system without utilizing filters and then we collected CT images using the SPECT-CT system (Symbia T2, Siemens). We reconstructed the location and energy of the Na^131^I radioactive sources based on hybrid spectral CLT. Our results showed that there was a very good linear correlation between the reconstructed source energy and the activity of the Cerenkov luminescent source (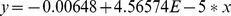 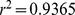).(TIF)
